# Gilt Vaccination with a Mixed Administration of a PRRS MLV and a PPV1 Subunit Vaccine Protects against Heterologous PRRSV1 Infection and Prevents Detrimental Effects on Piglet Performance

**DOI:** 10.3390/v12080789

**Published:** 2020-07-23

**Authors:** Beatriz Garcia-Morante, Rachel Friedrich, Troy Kaiser, Christian Kraft, Philip Bridger, Marta Noguera

**Affiliations:** 1 Centcinc Coworking, C, Montserrat de Casanovas 105, 08032 Barcelona, Spain; 2 Veterinary Diagnostic and Production Animal Medicine, College of Veterinary Medicine, Iowa State University, 1800 Christensen Drive, Ames, IA 50011, USA; rachfred@iastate.edu; 3 Boehringer Ingelheim Vetmedica Inc., 2621 North Belt Highway, St. Joseph, MO 64506, USA; troy.kaiser@boehringer-ingelheim.com; 4 Boehringer Ingelheim Veterinary Research Center GmbH & Co. KG, Bemeroder Straβe 31, 30559 Hannover, Germany; christian.kraft@boehringer-ingelheim.com; 5 Boehringer Ingelheim Vetmedica GmbH, Binger Str. 173, 55216 Ingelheim, Germany; philip.bridger@boehringer-ingelheim.com

**Keywords:** porcine reproductive and respiratory syndrome virus, porcine parvovirus, modified live virus vaccine, subunit vaccine, combined vaccine, efficacy, onset of immunity, duration of immunity

## Abstract

The efficacy of the combined administration of a porcine reproductive and respiratory syndrome (PRRS) modified live virus (MLV) vaccine and a porcine parvovirus 1 (PPV1) subunit vaccine in gilts was addressed in two experiments. Experiment A aimed to establish a 4-week onset of immunity (OOI). Gilts were randomly distributed in three treatment groups: non-vaccinated control animals (group 1), animals vaccinated with the combined vaccine (group 2), and a third group that consisted of animals vaccinated with the PRRS MLV vaccine alone (group 3). Four weeks after the first vaccination, gilts were challenged with a heterologous PRRS virus 1 (PRRSV1) and euthanized three weeks after. Besides this, experiment B pursued a 17-week duration of immunity (DOI). In this case, gilts were distributed in the same treatment groups, but for the third group, which consisted of non-vaccinated, non-challenged animals were used instead. For the DOI assessment, gilts were artificially inseminated 4 weeks after the first vaccination, challenged at day 90 of gestation, and followed up, together with their offspring, until day 20 post-farrowing. Serology and viremia post-challenge were determined in gilts from both experiments, while farrowing and piglet performance were only evaluated in experiment B. Overall, the combined vaccine helped to protect gilts from viremia post-challenge and, consequently, to prevent PRRS clinical symptoms and diminish the proportion of piglets infected congenitally or early in life. The combined vaccine also elicited a significant improvement in piglet survival rate and growth performance until weaning. The present results reveal efficacy and lack of interference of the mixed use of the tested vaccines against PRRSV1 infection, with at least 4-week OOI and 17-week DOI.

## 1. Introduction

Porcine reproductive and respiratory syndrome (PRRS) is considered to be one of the most important diseases affecting intensive pig production worldwide as it has a significant economic impact across breeding herds and growing pig populations [1,2,3]. When the PRRS virus (PRRSV) enters a herd, it usually becomes endemic and can circulate in the farm for several years. The maintenance of the infection within a farm is mostly due to the presence of animals with long-term infection and the constant availability of susceptible pigs [4]. Thus, preventing infection and transmission of the virus is essential in reducing its negative impact. The main features of most PRRSV-affected breeding herds consist of late-term abortions and the delivery of late-term dead fetuses and weak pigs [5]. Apart from PRRSV, many other viruses have been reported to affect porcine reproductive performance, including the porcine parvovirus genotype 1 (PPV1), recently named *Ungulate protoparvovirus* 1 [5,6,7]. Outstandingly, vaccination is considered as the principal method to control and treat PRRSV and PPV1 infections and, therefore, vaccination strategies against both infectious agents are routinely applied in breeding herds worldwide. 

While vaccination programs are crucial for swine producers to preserve herd health, simplifying immunization schedules by combining multiple vaccines may represent numerous positive effects, including, but not limited to, reduced administration costs and time, convenience of the user, and better animal welfare. Altogether, this promotes the development of combined vaccines, although the formulation of multivalent vaccines is often challenging due to putative immune interference. For instance, vaccination with a PRRS modified live virus (MLV) vaccine was reported to decrease the efficacy of a *Mycoplasma hyopneumoniae* bacterin [8]. Henceforth, combined vaccines may only be used if their efficacy and safety in combination has been proven. In this context, the safety and long-term immunity of the combined administration of a PRRS MLV vaccine and an inactivated bivalent vaccine against PPV1 and *Erysipelothrix rhusiopathiae* has been recently disclosed [9]. In addition, we also have reported on the safety and compatibility of the combined administration of a PRRS MLV vaccine (ReproCyc^®^ PRRS EU) and a novel subunit vaccine based on the viral protein (VP) 2 of PPV1 (ReproCyc^®^ ParvoFLEX) [10]. Nevertheless, data concerning the efficacy afforded by such vaccine combination are yet to be published. Overall, evaluation of the efficacy of a combined vaccine requires demonstration that mixing the vaccines’ components does not negatively affect the onset and duration of immunity (OOI and DOI, respectively) and does protect against controlled challenge conditions, as established for each individual vaccine.

Both ReproCyc^®^ PRRS EU and ReproCyc^®^ ParvoFLEX vaccines have been previously demonstrated to be safe and efficacious in several published works [10,11,12,13,14]. In this paper, we expand on our studies on the European-derived PRRS MLV vaccine by determining the OOI and DOI when mixed with the novel PPV1 subunit vaccine. Immunogenicity of the combined vaccine was then established in the face of a disease-inducing heterologous PRRS virus 1 (PRRSV1) challenge in breeding-age gilts. To achieve this objective, two experiments (A and B) were conducted to assess differences between vaccinated and unvaccinated animals. In both experiments, the outcome parameters in gilts were the percentage of viremic and antibody positive animals as well as the reduction of viral loads post-challenge. The reproductive performance of gilts and parameters in piglets such as viremia at birth, clinical observations, survival rate, and average daily weight gain (ADWG) were uniquely assessed in experiment B, which assessed the DOI.

## 2. Materials and Methods 

### 2.1. Animals and Housing

Two experiments were conducted to establish the OOI (experiment A) and DOI (experiment B) with 36 and 37 commercial mixed-breed, non-pregnant, breeding-age gilts, respectively. All gilts enrolled in the studies were seronegative for PPV1 by hemagglutination inhibition (HI) and for PRRSV by enzyme-linked immunosorbent assay (ELISA) as well as negative for PPV1 and PRRSV viremia, as tested by gel-based polymerase chain reaction (PCR) and reverse transcription quantitative PCR (RT-qPCR), respectively. In each experiment, gilts were randomly assigned to one of three treatment groups and housed by treatment in separate rooms at Veterinary Resources, Inc. (VRI; Ames, IA, USA). All rooms were biohazard level 2 (BL2) compliant, high efficiency particulate air (HEPA)-filtered, and mechanically ventilated with thermostat-regulated temperature control. In all cases, gilts were transported to the center prior to initiation of the study, on study day (SD) 0, allowing the animals a period to acclimate. Gilts were fed a non-medicated commercial ration that was produced locally and appropriate for their size, age, and condition according to acceptable animal husbandry practices for the region. Water was available ad libitum. Experiment B was terminated 20 days after farrowing, and piglets from the enrolled primiparous female pigs were offered commercially prepared, non-medicated pre-starter ration *ad libitum* from the second week of life onwards. This study was approved by the Veterinary Resources Inc. Institutional Animal Care and Use Committee (IACUC 2015065, approved on the 2 October 2015).

### 2.2. Experimental Design

#### 2.2.1. Experiment A: Onset of Immunity

The design of experiment A is summarized in Table 1. Gilts were randomly distributed in three treatment groups. Group 1A-control product (CP; *n* = 12) received phosphate buffered saline (PBS) twice in a 3-week interval (SD 0 and 21). Group 2A-PRRSV1+PPV1 (*n* = 12) received the European-derived PRRS MLV vaccine mixed with the novel PPV1 subunit vaccine at SD 0 and the PPV1 subunit vaccine alone at SD 21. The PRRS MLV vaccine was administered alone and uniquely at SD 0 in those animals belonging to group 3A-PRRSV1 (*n* = 12). Challenge with a heterologous PRRSV1 strain was carried out 4 weeks after the first treatment administration (SD 28). Three weeks after challenge (SD 49), all gilts were euthanized, and the study terminated.

#### 2.2.2. Experiment B: Duration of Immunity

The design of experiment B is summarized in Table 1. Gilts were arbitrarily assigned to three groups. Group 1B-CP and group 2B-PRRSV1+PPV1 received their respective treatments at the same time points (SD 0 and 21) as described above for experiment A. In this case, the third group included non-treated, non-challenged strict control (SC) animals. Gilts in experiment B were estrus synchronized by administration of 6.8 mL (15 mg altrenogest) per gilt by top-dressing Matrix^®^ (Merck Animal Health, Madison, NJ, USA) on a portion of each gilt’s daily feed for 14 consecutive days (from SD 8 to SD 21). Four to 9 days after completion of treatment (from SD 25 to SD 30), gilts were artificially inseminated, and those confirmed pregnant were challenged at approximately 90 days of gestation (SD 118). Primiparous sows and their offspring were followed up for 20 days after farrowing, when experiment B concluded.

### 2.3. Primary Outcome Parameter and Justification for Number of Animals

In both experiments, the primary outcome parameter for vaccine efficacy evaluation was the proportion of viremic gilts as well the PRRSV load post-challenge. For calculations of animal numbers, a relevant difference was set at a reduction of 1.0 log10 genome equivalent (GE)/mL/day. Based on previous studies, the standard deviation was expected to be 0.6 log10 GE/mL. The comparison-wise type I error alpha was adjusted to 0.025 in order to keep the experiment-wise type I error alpha of 0.05. Ten animals per treatment group were expected to provide approximately 80% power to detect a relevant difference of 1.0 log10 GE/mL/day between the vaccinated and the control groups for a two-sided Wilcoxon Mann–Whitney test. Henceforth, a total number of 12 and 16 animals per group in experiment A and B, respectively, were used in order to compensate for possible dropouts. 

### 2.4. Vaccination and Control Product Administration

The PRRS MLV vaccine used was the licensed ReproCyc^®^ PRRS EU (Boehringer Ingelheim Vetmedica GmbH, Ingelheim am Rhein, Germany), which contains an adjuvanted live attenuated PRRSV1 isolate of German origin from 2002. The vaccine strain shares 90.4% sequence homology in the ORF5 gene locus to the reference strain Lelystad [12], and its complete genome is available in GenBank (accession No. KT988004). For the PPV1 subunit vaccine ReproCyc^®^ ParvoFLEX (Boehringer Ingelheim Vetmedica GmbH, Ingelheim am Rhein, Germany), a manufacturing technology of baculovirus expression system is used to express the viral protein 2 of an isolate from 2001 (strain 27a) [14]. In this case, the PPV1 subunit vaccine was formulated to contain the maximum antigen content per dose to perceive, if any, interference between antigens when mixing vaccines. For the combination of both vaccines, the freeze-dried cake of the PRRS MLV vaccine was reconstituted with the PPV1 subunit vaccine, a liquid suspension partly comprised of a sterile carbomer adjuvant solvent, namely ImpranFLEX^®^ (Boehringer Ingelheim Animal Health USA Inc., St. Joseph, MO, USA). The PRRS MLV vaccine rehydration was performed such that the final targeted inclusion level of PRRSV1 was the minimum commercial range per dose, thus demonstrating vaccine compliance in a minimum vaccine dose scenario. Additionally, sterile PBS was used as a placebo control product. All treatments were administered intramuscularly (i.m.) into the neck caudal to the ear base and at a volume of 2 mL per dose. The initial dose administered (SD 0) was applied in the right side of the neck, whereas second injections (SD 21), when applicable, were given in the left side of the neck.

### 2.5. Challenge Inoculation

Challenge of animals was conducted at different time points in experiments A and B (Table 1). For the OOI assessment (experiment A), challenge was carried out 4 weeks after the first vaccination (SD 28), whereas for the DOI determination (experiment B), challenge was conducted at approximately day 90 of gestation (SD 118). In all cases, animals were challenged with the virulent, low-passage European PRRSV1 isolate 190136, propagated to a titer of 4.75 log10 median tissue culture infectious dose (TCID)_50_/mL. The origin of the challenge isolate as well as its genetic relationship with the vaccine master seed isolate have been formerly described [11,13]. Briefly, the PRRSV1 challenge isolate was originally derived from the lung tissue of a newborn piglet during a PRRS outbreak in Germany, in April 2004, and exhibits less than 87% genetic identity to the PRRS MLV vaccine isolate within the complete genome (or 88%, based on ORF5/ORF7 data). In both experiments, all gilts were inoculated with 2 mL of challenge material per nostril and 2 mL i.m. in the left neck—a total of 6 mL of challenge material per gilt.

### 2.6. Blood Collection and Processing

In experiment A, venous whole blood was collected from gilts 2 and 3 weeks after first treatment administration (SD 14 and 21) and just before PRRSV1 challenge (SD 28). After challenge, bleeding was performed every 3 to 4 days at SD 31, 35, 38, 42, and at termination of the study (SD 49). Samples obtained until challenge were used for serologic testing, whereas samples from challenge onwards were processed for viremia. In experiment B, blood was collected from gilts weekly after injection of the first treatment at SD 7, 14, and 21. Then, throughout pregnancy, gilts were sampled at approximately 28 (SD 56), 56 (SD 84), 90 (SD 118), 97 (SD 125), and 104 (SD 132) days of gestation. In this case, all samples were used for serologic analysis, and those obtained from challenge onwards were used for viremia assessment. Venous whole blood was also collected from each live-born piglet within 24 h of farrowing and, if possible, from each non-viable piglet (dead or mummy). If blood could not be collected, lung tissue was attained using sterile material. All blood samples were allowed to clot at room temperature and centrifuged, and serum was then correspondingly harvested for further laboratory testing.

### 2.7. Serologic and Viremia Testing 

Porcine parvovirus 1 and PRRSV pre-screening were performed at the Iowa State University-Veterinary Diagnostic Laboratory (ISU-VDL; Ames, IA, USA). Once the experiments started, the serum samples held at 2 °C to 8 °C were externally tested for PRRSV antibodies at the ISU-VDL with a commercially available indirect ELISA kit (IDEXX HerdChek PRRS X3 ELISA, IDEXX Laboratories, Westbrook, ME, USA). Results were reported as negative (sample to positive (S/P) ratio of <0.4) or positive (S/P ratio ≥ 0.4). Viremia was assessed from frozen sera (−70 ± 10 °C), which were shipped and tested at Boehringer Ingelheim Veterinary Research Center (BIVRC) GmbH & Co. KG (Hannover, Germany) for PRRSV RNA detection by RT-qPCR, as described elsewhere [15,16]. Briefly, the 2× *Taq*Man Universal PCR Kit with MultiScribe™ RT-Mix (Applied Biosystems; Foster City, CA, USA), the EU6-MGB (CTGTGAGAAAGCCCGGAC) probe, and the primers EU6-343f-plus (GTRGAAAGTGCTGCAGGYCTCCA; sense) and EU6-462r-plus (CACGAGGCTCCGAAGYCCW; antisense) were used. Genome equivalence was determined with use of the plasmid standard, and results were reported as log10 GE/mL. In the same manner, all piglet sera, fluid, or lung tissue samples from experiment B were also sent to BIVRC for PRRSV RNA detection by means of RT-qPCR. It is worth mentioning that, for statistical purposes, a RT-qPCR result of “not detected” was assigned a value of 0.0 log10 GE/mL while a result of “positive” but unquantifiable was assigned a value of 3.0 log10 GE/mL.

### 2.8. Farrowing Performance and Survival Rate of Piglets 

In experiment B, farrowing data were recorded for each primiparous sow. The day of farrowing for each animal was defined as the day that the first piglet was delivered. At the time of farrowing, each piglet was classified into one of five categories: “mummy”, “stillborn”, “crushed”, “weak”, or “healthy”. Piglets “alive” at farrowing were calculated as (healthy + weak + crushed)/total number of piglets farrowed. Relative frequencies of piglets per category were calculated in relation to the total number of piglets at farrowing per gilt and used as single values for the comparison between groups. Similarly, the percentage of piglets per litter alive at 20 days post-farrowing was calculated in relation to the number of piglets alive at farrowing minus the number of crushed piglets. 

### 2.9. Observations and Measurement in Piglets

Piglets from experiment B were observed for clinical signs of general health starting the day after farrowing until 20 days of life. Observations were performed as described previously [15]. Briefly, each piglet was visually examined in the pen before handling and was scored 0 to 2 for severity of clinical signs including “coughing”, “abnormalities of respiration”, and “behavior”. The percentages of piglets per litter with at least one abnormal finding (score > 0) were calculated and used as single values for the comparison between groups. Regardless of health status, individual body weights in kg were collected using a calibrated scale (accuracy: ±0.02 %) at the day of farrowing as well as prior to the end of the study, at either the time of death or the conclusion of the study (20 days post-farrowing). 

### 2.10. Analysis of Data

All data were imported into SAS 9.4 or a higher version for management and evaluation, while graphs were performed using Graph Pad Prism 8 software. In both experiments, data were analyzed assuming a completely random design structure, although groups were housed in separate rooms throughout the study to prevent cross-contamination of treatments. Given that animals of different treatment could not be commingled, analyses of gilt outcomes were performed considering “gilt” as the experimental unit, and analyses of “piglet” outcomes (experiment B) were performed considering “litter” as the experimental unit. Data were summarized using descriptive statistics based on the type of variable with a 95% confidence interval (CI). The efficacy of vaccines was established as the lack of significant differences between the vaccinated groups (2A-PRRSV1+PPV1, 3A-PRRSV1 and 2B-PRRSV1+PPV1) and the corresponding unvaccinated negative control groups (1A-CP and 1B-CP). In experiment B, group 3B-SC was excluded from statistical analyses. 

Frequency tables of positive ELISA and RT-qPCR results from gilts were generated and differences between groups were evaluated using the Fisher‘s exact test. In the case of piglets, the percentages of RT-qPCR positives per litter were used as single values for the comparison between groups by the Wilcoxon Mann–Whitney test. For the assessment of viral loads, the mean and median RT-qPCR values (log10 GE/mL) per gilt and piglet, respectively, were calculated and used for the comparisons between groups by the Wilcoxon Mann–Whitney test. To evaluate farrowing performance, absolute frequencies per gilt of total number, living, healthy, weak, stillborn, mummified, and crushed piglets at farrowing and living piglets at 20 days post-farrowing were determined and evaluated using the Wilcoxon Mann–Whitney test. The ADWG was calculated per piglet and 20 days post-farrowing period. Differences between treatment groups were tested by analysis of variance (ANOVA) and subsequent *t*-tests. The least squares mean (LSM) of the groups and differences between them with 95% CI were derived from the ANOVA. For the analysis of body weight at 20 days post-farrowing and ADWG, the weight recorded at farrowing was used as a covariate. Lastly, the percentages of piglets per litter with at least one clinical observation (score > 0) were calculated and used as single values for the comparison between groups by the Wilcoxon Mann–Whitney test. In both experiments, all tests between groups were designed as two-sided tests on differences, and differences were considered as statistically significant only if *p* ≤ 0.05. 

## 3. Results

### 3.1. Proportion of Viremic Gilts after Challenge

**Figure 1:**
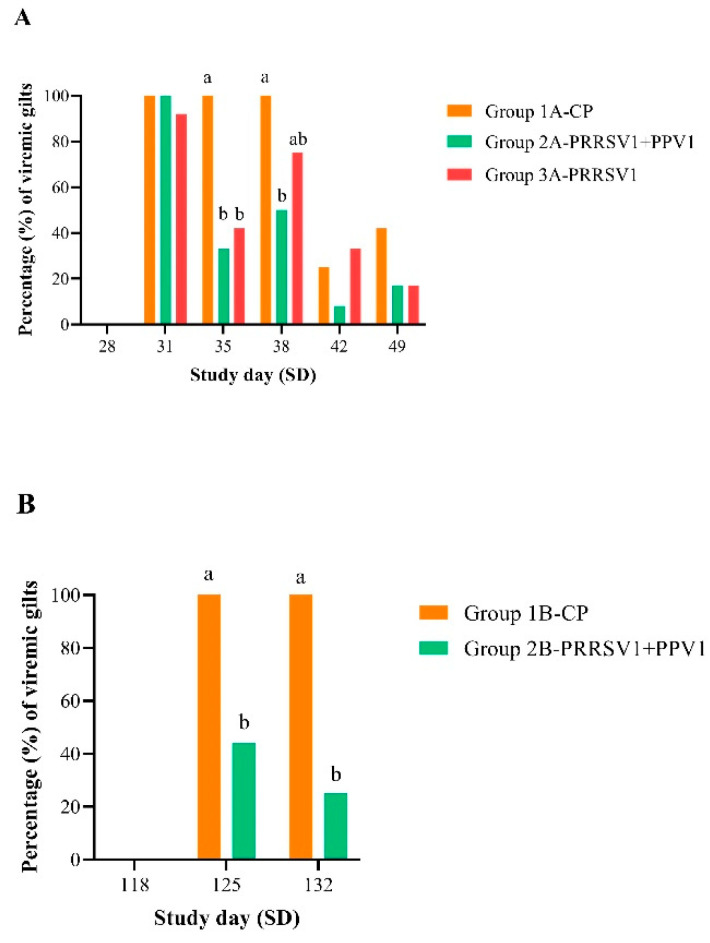
Proportion (%) of PRRSV RNA-positive gilts from challenge onwards in experiment A (**A**) and B (**B**). PRRS viral RNA load was assessed in sera using RT-qPCR. Frequency tables of positive results were generated and differences between groups were tested by Fisher’s exact test. Different letters indicate statistical significance of differences between groups.

The percentage of gilts that were positive for PRRSV RNA from challenge onwards in both experiments is shown in Figure 1. It is important to mention that all control gilts (groups 1A- and 1B-CP) remained negative for PRRSV RNA until challenge. In experiment B, the non-vaccinated, non-challenged gilts (group 3B-SC) also remained negative throughout the study. In experiment A (OOI assessment), nearly all gilts tested positive for PRRSV RNA by 3 days after challenge (SD 31), as only one gilt out of 12 was negative in group 3A-PRRSV1. Thereafter, the percentage of PRRSV RNA-positive gilts declined to 33% and 42% in the mixed (2A-PRRSV1+PPV1) and single (3A-PRRSV1) vaccination groups, respectively. Indeed, both groups had significantly lower percentages of viremic gilts than the challenge control group 1A by SD 35 (*p* ≤ 0.01). Ten days after challenge (SD 38), a second increase in viremia was observed in both vaccinated groups; six and nine gilts out of 12 were PRRSV RNA-positive in groups 2A-PRRSV1+PPV1 and 3A-PRRSV1, respectively. Nonetheless, the percentage of viremic gilts in group 1A-CP was still significantly higher when compared to the mixed vaccinated group 2A (*p* ≤ 0.01). Afterwards, the percentage of viremic gilts in the challenge control group 1A fell considerably, and there were no significant differences in the percentages of PRRSV RNA-positive gilts in comparison with the vaccinated groups, 2A and 3A. In experiment B (DOI assessment), 100% of the 16 control gilts were PRRSV qPCR-positive at 7 (SD 125) and 14 (SD 132) days post-challenge, while 44% (7 out of 16) and 25% (4 out of 16) of the mixed vaccinated gilts were PRRSV qPCR-positive on SD 125 and SD 132, respectively. Therefore, the proportion of viremic gilts was significantly reduced in the mixed vaccinated group 2B at both time points when compared to the challenge control group 1B (*p* ≤ 0.0001).

### 3.2. Viral Loads in Gilts after Challenge

**Figure 2:**
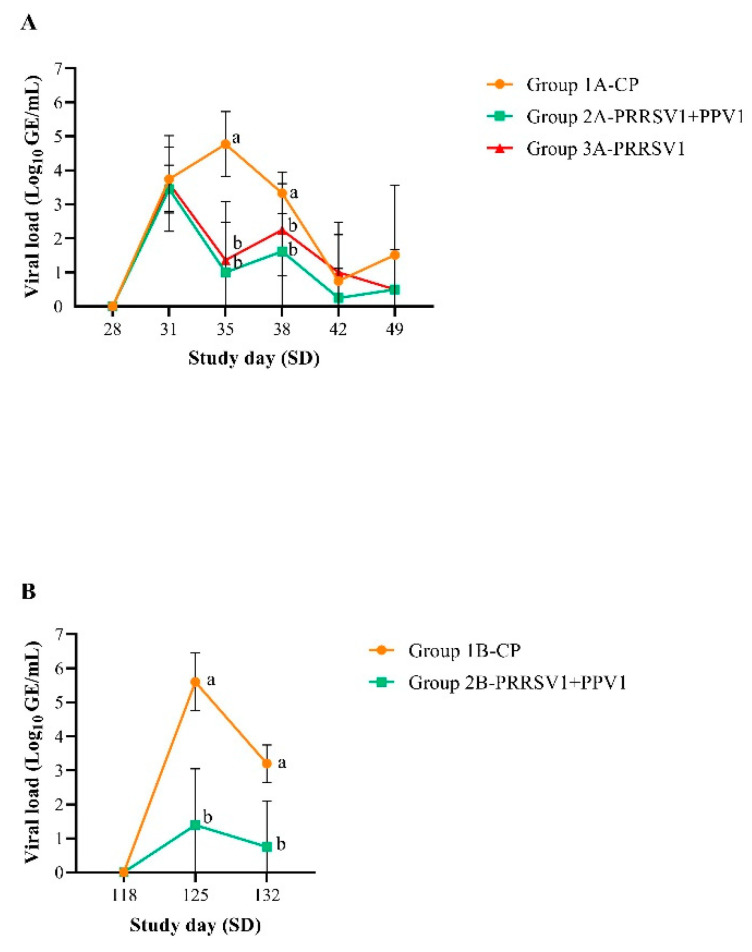
PRRSV viral RNA load (mean ± standard deviation) in gilts from challenge onwards in experiments A (**A**) and B (**B**). PRRS viral RNA load was assessed in sera using RT-qPCR. Generated data in log10 GE/mL were used for comparisons of mean values between groups using the Wilcoxon Mann–Whitney test. For statistical purposes, a RT-qPCR result of “not detected” was assigned a value of 0.0 log10 GE/mL, while a result of “positive” but unquantifiable was assigned a value of 3.0 log10 GE/mL. Different letters indicate statistical significance of differences between groups.

Post-challenge gilt PRRSV viral load results in serum from both experiments are presented in Figure 2. In experiment A, the viral RNA load increased between challenge and the following 3 days (SD 31) in all groups (i.e., mean above 3.0 log10 GE/mL). However, both vaccinated groups, 2A and 3A, had significantly lower mean viral RNA loads compared with the challenge control group 1A at 7 (SD 35; *p* ≤ 0.01) and 10 (SD 38; *p* ≤ 0.05) days after challenge. Indeed, the viral load dynamics in both vaccinated groups were similar, without statistically significant differences in the viral load at any time point. In parallel, vaccinated gilts from experiment B (group 2B-PRRSV1+PPV1) had significantly lower mean PRRSV viral loads at 7 (SD 125; *p* ≤ 0.0001) as well as at 14 (SD 132; *p* ≤ 0.0001) days post-challenge when compared to their counterparts from group 1B-CP.

### 3.3. Gilt Serology

**Figure 3:**
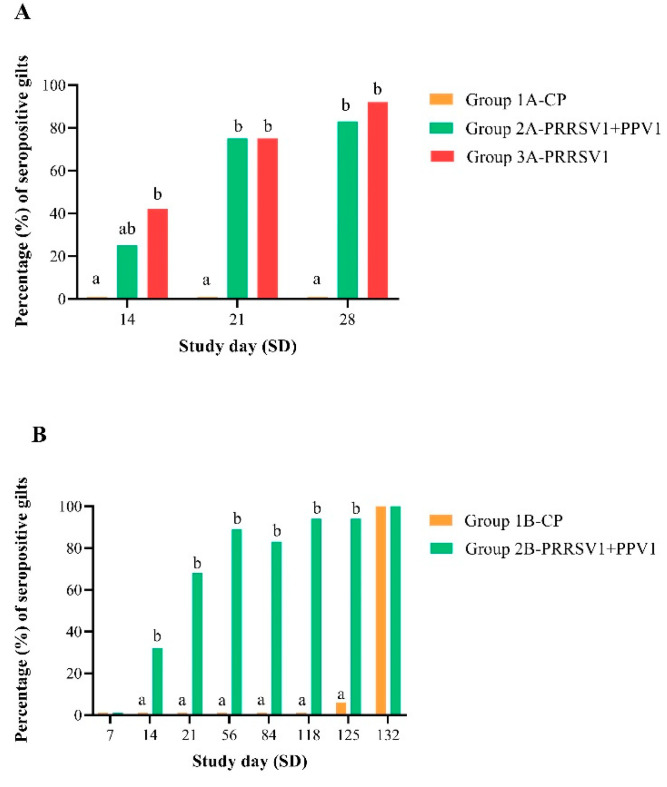
Proportion (%) of seropositive gilts to PRRSV at different time points in experiments A (**A**) and B (**B**). Sera were tested for PRRSV antibodies by means of a commercially available indirect ELISA kit. Frequency tables of positive results were generated and differences between groups were tested by Fisher’s exact test. Different letters indicate statistical significance of differences between groups.

Porcine reproductive and respiratory syndrome virus serology results from experiments A and B are depicted in Figure 3. As expected in both experiments, all control gilts (groups 1A-CP and 1B-CP) were PRRSV seronegative up to the day of challenge; SD 28 and SD 118 in experiments A and B, respectively. In addition, all samples of group 3B-SC tested negative throughout the study period. In experiment A (OOI assessment), the PRRSV1 vaccinated group 3A showed a statistically significantly higher percentage of seropositive gilts from 2 weeks after vaccination (SD 14; *p* ≤ 0.05) to challenge (SD 28; *p* ≤ 0.0001), as compared to the negative control group 1A. In the case of the mixed vaccinated group 2A, a statistically significantly higher percentage of seropositive gilts appeared 3 weeks after vaccination (SD 21; *p* ≤ 0.0001) and extended to challenge (SD 28; *p* ≤ 0.0001) when compared with gilts from group 1A-CP. Hence, 83% and 92% of gilts were seropositive at challenge in groups 2A-PRRSV1+PPV1 and 3B-PRRSV1, respectively. In experiment B (DOI assessment), the proportion of PRRSV ELISA positive results in gilts from group 2B was significantly higher from two weeks after vaccination (SD 14; *p* ≤ 0.05) until approximately day 97 of pregnancy (SD 125; *p* ≤ 0.01) when compared to control gilts from group 1B, consistent with a serological response to vaccination. By two weeks post-challenge (SD 132), the percentage of seropositive gilts reached 100% in both groups, increasing steeply in group 1B-CP as a response to challenge. By then, there were no significant differences in the percentages of PRRSV antibody positive gilts between groups.

### 3.4. Gilt Reproductive Performance and Piglet Survival Rate

A gilt from group 2B-PRRSV1+PPV1 farrowed prematurely (on SD 125, approximately two weeks prior to projected farrowing date) and delivered a litter of 15 piglets, mostly stillborn and a few alive but weak and non-viable piglets. This gilt was viremic (positive but unquantifiable by RT-qPCR) at farrowing; however, all the offspring but one piglet (6.35 log10 GE/mL) were negative for PRRSV by RT-qPCR. The gilt recovered without incident within a few days; however, farrowing as well as piglet data from this litter were not considered in the analyses. 

**Figure 4:**
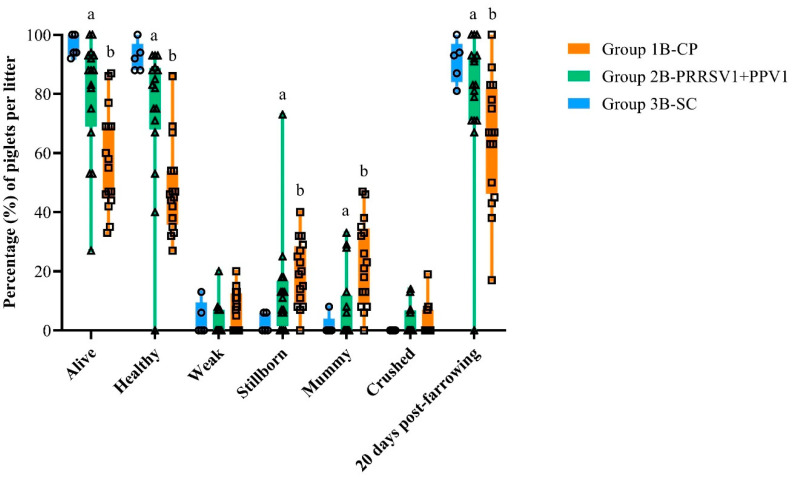
Box plots of proportion (%) of piglets per litter in each reproductive performance category and living piglets at the end of experiment B (20 days post-farrowing). The boxes extend from the 25th to 75th percentiles and the whiskers cover the smallest % value up to the largest. Absolute frequencies per litter of living, healthy, weak, stillborn, mummified, and crushed piglets at farrowing and living piglets at 20 days post-farrowing were determined and evaluated using the Wilcoxon Mann–Whitney test. Group 3B-SC was excluded from statistical analyses. Different letters indicate statistical significance of differences between groups.

For all the other primiparous sows from experiment B, farrowing data were recorded and the proportion of piglets per litter in each farrowing performance category as well as alive at 20 days post-farrowing are represented in Figure 4. The number of piglets farrowed per litter was not significantly different between treatment groups; this was 15.2 and 14.8 in group 1B-CP and 2B-PRRSV1+PPV1, respectively. However, vaccinated gilts from group 2B had a statistically significantly higher percentage of total live-born piglets (category “alive”) per litter when compared to control gilts from group 1B (*p* ≤ 0.01). They also had an additional advantage in the percentage of healthy piglets per litter (category “healthy”; *p* ≤ 0.01) and, consequently, a significantly lower percentage of mummy (category “mummy”; *p* ≤ 0.0001) and stillborn piglets (category “stillborn”; *p* ≤ 0.05) per litter than control gilts. Although not statistically significant compared to control gilts, there was also a lower percentage of weak born piglets per litter farrowed by vaccinated gilts. Lastly, piglets born from vaccinated gilts had a statistically significantly better survival rate at 20 days post-farrowing than piglets born from control gilts (*p* ≤ 0.0001). 

### 3.5. Viral Loads and Proportion of Viremic Piglets

Proportion of positive piglets for PRRSV by RT-qPCR per litter and piglet viral load results are presented in Table 2. No virus was detected in strict control piglets of group 3B. At birth, the mean proportion of PRRSV positive piglets per litter from vaccinated gilts was statistically significantly lower compared to litters from the challenged control gilts (*p* = 0.005). Likewise, piglets in group 2B-PRRSV1+PPV1 had a statistically significantly lower median PRRSV viral load than piglets in group 1B-CP (*p* = 0.0025). 

### 3.6. Piglet Clinical Observations

The percentages of piglets per litter with at least one abnormal observation (score > 0) throughout the observation period were compared between groups and are shown in Table 3. The effect of vaccination was readily apparent, as the mean percentage of piglets per litter with at least one abnormal finding (score > 0) in the control group 1B was statistically significantly higher compared to the vaccinated group 2B (*p* ≤ 0.0001).

### 3.7. Piglet Average Daily Weight Gain

Mean body weight and ADWG of piglets from experiment B are summarized in Table 4. The difference in the mean body weight of piglets at birth between treatment groups was not statistically significant. However, piglets in the vaccinated group 2B had statistically significantly higher body weights at 20 days post-farrowing as well as an increased ADWG compared to control piglets from group 1B (*p* ≤ 0.01). Such variation between groups led to an LSM positive difference of 50 g gained daily per piglet from vaccinated dams compared to control piglets throughout the observation period of 20 days post-farrowing. 

## 4. Discussion

Although various viral pathogens of swine may affect reproductive performance, PRRSV and PPV1 are considered among the major causes [5,6]. Vaccination programs against both pathogens are routinely implemented in breeding herds, and, therefore, a potential combination vaccine will minimize animal handling and reduce caretakers’ daily workloads. Nevertheless, vaccines may only be administered together if their efficacy and safety in combination has been proven. Thus, the aim of this study was to evaluate the efficacy of the combined administration of a PRRS MLV vaccine and a PPV1 subunit vaccine in gilts after challenge with a heterologous and pathogenic PRRSV1 strain under controlled laboratory conditions. The safety and efficacy of each vaccine applied individually have been shown in previous studies in the target species [11,12,13,14]. Moreover, the use of this combination has been already reported in the field; the safety and lack of antigen interference of the vaccines were proven in a farm where the circulation of a wild type PRRSV1 virus was confirmed [10]. To the best of our knowledge, this is the first report of the efficacy of the combined use of a PRRS MLV and a PPV1 subunit vaccine. 

A set of two studies was outlined to demonstrate efficacy against PRRSV1 of the associated use of the abovementioned vaccines. Experiment A was designed to establish a 4-week OOI while experiment B was designed to demonstrate lack of interference at 17 weeks (DOI). Additionally, the efficacy of the combined vaccine against PRRSV1 was evaluated by assessing piglet performance during lactation in experiment B. Both experiments were conducted according to the guideline EMA/CVMP/IWP/594618/2010, “requirements for combined vaccines and associations of immunological veterinary medicinal products (IVMPs)”. This document outlines items to be considered and the data requirements in relation to marketing authorization applications where an association between two or more different IVMPs is claimed. This text does not explicitly mention the need for a monovalent group to prove efficacy nor safety. Here, a monovalent group (i.e., 3A-PRRSV1) was only included in experiment A because a shorter OOI needed to be proven as compared to the 5-week OOI established during the development of the ReproCyc^®^ PRRS EU vaccine. 

All gilts were free from PRRSV, as demonstrated by the absence of PRRSV RNA in their serum followed by seronegative ELISA test results before initiation of experiments. In addition, RT-qPCR results indicated that all gilts were free from PRRSV on the day of challenge. These data confirm that no PRRSV exposure or cross-contamination among vaccinated and control groups occurred up to the point of challenge, validating subsequent findings of this report. In addition, a group of non-vaccinated, non-challenged control gilts was included in experiment B (i.e., group 3B-SC); these remained seronegative and viremia negative throughout the study, which reinforces the previous statement. The lack of observed vaccine-induced viremia by challenge was probably due to the resolution of viremia before 14 days post-vaccination, as previously described for the used PRRS MLV1 vaccine [11]. 

The adverse effects of PRRSV are primarily a consequence of acute viremia post-infection [17]. Therefore, reducing viremia is important in PRRSV control programs. In addition, reduced viremia leads to reduced shedding from infected pigs and thus reduced virus transmission [18,19]. A key objective of both vaccination challenge experiments was, indeed, to demonstrate a statistically significant reduction in the proportion of viremic animals and viral loads for vaccinated gilts compared to control gilts post-challenge. Either the single immunization with the PRRS MLV1 vaccine or the combination with the PPV1 subunit vaccine significantly reduced PRRSV viremia (i.e., lower viral RNA load and fewer PRRSV RNA-positive gilts) by 7 days post-challenge. Reduction of viremia was statistically significant at least until 14 days after challenge in gilts vaccinated with the combined product. These findings were in alignment with previously reported data on the reduced post-challenge viremia in a PRRS MLV vaccination context [11,13] and suggest a lack of interference with the PPV1 subunit vaccine, corroborating previously reported data [10].

Porcine reproductive and respiratory syndrome virus-specific antibodies appear at approximately 14 days and peak around 28 days after vaccination with a PRRS MLV vaccine [20]. Accordingly, consistent seroconversion following vaccination was observed in both experiments. In experiment A, PRRSV ELISA serology supported a significant increase in positive results, starting at day 21 post-vaccination with the mixed vaccine and at day 14 post-vaccination with the PRRS MLV vaccine alone. This finding could suggest a certain degree of delay in seroconversion when the combined product is applied. However, the latter was not reproduced in experiment B, as a significant increase in seropositivity was already observed at 14 days post-vaccination with the mixed vaccine. Seroconversion was not complete in either the single-vaccination or mixed-vaccination group by day of challenge. However, challenge-induced viremia reduction was not linked to seropositivity at challenge in any of the studies. Incomplete seroconversion alongside full protection from PRRS has already been described in other studies and most likely reflects the relevant role of the cell-mediated immunity in PRRSV clearance from the blood as well as the variability in immune responses between animals [11,21,22].

Piglets born to dams infected with PRRSV may suffer from increased weakness at birth, decreased growth rates, increased susceptibility to respiratory infections, and higher pre-weaning mortality [5,23]. They can harbor the virus for several months and can contribute to the spread of the infection in the following productive stages [4]. As vaccinating dams helps prevent viral shedding and reduces both horizontal and vertical PRRSV transmission, vaccination before breeding is considered to be of value for decreasing PRRS detrimental effects [24]. In support of the above, several studies have demonstrated that the use of European-derived PRRS MLV vaccines significantly improves reproductive performance at farrowing and the number of live pigs at weaning [23,25,26]. Moreover, previous studies demonstrated that a single dose of ReproCyc^®^ PRRS EU was successful in protecting farrowing gilts and sows and their offspring from PRRS [12,13]. Experiment B was designed to evaluate whether such effectiveness was maintained when mixed with a PPV1 subunit vaccine (ReproCyc^®^ ParvoFLEX). Gilts from the mixed vaccination group had a significant advantage over the challenge control gilts in the assessment of reproductive performance; vaccinated gilts farrowed on average 3.7 more healthy live piglets per litter than control gilts. On this matter, there were significant beneficial differences in the mixed vaccinated group compared to the challenge control group for percentages of stillborn and mummified piglets per litter. Nonetheless, there was a gilt immunized with the combined vaccine that prematurely farrowed a large litter of stillborn and non-viable piglets. This gilt was viremic at farrowing (SD 125); however, all the offspring but one piglet were negative for PRRSV by RT-qPCR. While PRRSV1 infection due to the challenge cannot be ruled out as the cause of the premature parturition, the increased fetal stress secondary to the large litter should also be considered as a possible contributing factor.

Congenital infection was noted in piglets born to both vaccinated and unvaccinated gilts, though the viral load in serum as well as the number of viremic piglets were significantly lower in litters from gilts vaccinated with the PRRS MLV vaccine combined with the PPV1 subunit vaccine. These findings are consistent with previous studies that have reported that vaccinating gilts before breeding and subsequent challenge with PRRSV during gestation leads to a lack of transmission of the virus among littermates [27,28]. The latter is in line with the significantly milder clinical findings recorded in piglets from vaccinated dams compared to piglets from the control group, as well as with the statistically significant difference in mortality rates during lactation between groups of piglets. Moreover, piglets born to vaccinated primiparous sows gained weight at the same rate as piglets born to non-vaccinated, non-challenged control gilts (group 3B-SC), whereas piglets of the challenge control group grew significantly more slowly. In summary, the vaccination of gilts before breeding with the MLV vaccine against PRRSV mixed with the PPV1 subunit vaccine helped to prevent the reproductive failure caused by PRRSV1 infection during gestation. However, as formerly reported for PRRS MLV vaccines [24,28], the protection conferred by this combined product was partial as it could not completely prevent re-infection and transplacental infections caused by a heterologous PRRSV1 strain.

The primary objective of demonstrating the lack of interference for efficacy against PRRSV1 for the evaluated vaccine combination was met. As reported for other PRRS MLV vaccines, when applied alone, the combined vaccine helped to protect gilts from viremia and helped to reduce numbers of pre- and post-natal deaths and congenitally infected piglets [28]. Additionally, piglets born to vaccinated gilts with the mixed product had higher body weights and survival rates at weaning than those born to non-vaccinated control gilts [29]. Nevertheless, caution should be exercised when extrapolating the reported results across field conditions, as many factors in the field may influence the PRRSV dynamics of the infection and the protection conferred by the vaccines. Although safety and lack of antigen interference have been already observed in the field when using the PRRS MLV vaccine combined with the PPV1 subunit vaccine [10], efficacy against PPV1 under controlled conditions must be also addressed to ensure full compliance of the ReproCyc^®^ PRRS EU and ReproCyc^®^ ParvoFLEX mixture. A study focusing on the efficacy of the combined vaccine in pregnant gilts experimentally challenged with a heterologous PPV1 strain is still pending.

## 5. Conclusions

The results of this study demonstrate the efficacy and lack of interference of the mixed PRRS MLV and PPV1 subunit vaccines for both 4-week OOI and 17-week DOI against PRRSV1 heterologous challenge. The combined vaccination was effective in reducing PRRSV viremia and in reducing the detrimental effects that a virulent PRRSV1 infection can have in breeding female pigs and their offspring. These findings have important implications for herd management, as vaccination against both PRRS and porcine parvovirosis are regularly applied in breeding herds, and a combination of both vaccination programs could be highly convenient as an integral part of good management, particularly in endemic areas. 

## Figures and Tables

**Table 1:** Designs of experiment A and experiment B. The enrolled PRRSV naïve gilts were split into three treatment groups in each experiment. Experiment A was terminated 3 weeks after PRRSV1 challenge (SD 49). Gilts from experiment B were estrus synchronized, inseminated, and challenged at approximately 90 days of gestation (SD 118). In this case, the study was terminated 20 days after farrowing.

	No. of Animals	Treatment Groups	SD 0	SD 21	SD 28	SD 118
**Experiment A (OOI)**	12	1A-CP	PBS ^1^	PBS	Challenge	---
	12	2A-PRRSV1+PPV1	Mixed Vaccine ^2^	PPV1 subunit Vaccine ^3^	Challenge	---
	12	3A-PRRSV1	PRRS MLV Vaccine ^4^	---	Challenge	---
**Experiment B (DOI)**	16	1B-CP	PBS	PBS	---	Challenge
	16	2B-PRRSV1+PPV1	Mixed vaccine	PPV1 subunit vaccine	---	Challenge
	5	3B-SC	---	---	---	---

^1^ Phosphate buffered saline ^2^ ReproCyc^®^ PRRS EU combined with ReproCyc^®^ ParvoFLEX ^3^ ReproCyc^®^ ParvoFLEX ^4^ ReproCyc^®^ PRRS EU.

**Table 2:** Summary statistics for the proportion (%) of viremic piglets per litter (A) and piglet viral loads (Log10 GE/mL; B) at birth from experiment B. PRRS viral RNA load in sera or lung tissue was assessed using RT-qPCR. Generated data in log10 GE/mL were used for comparisons of median values between groups using the Wilcoxon Mann–Whitney test. Min. = minimum value, Max. = maximum value, STD = standard deviation, IQR = interquartile range, CI = confidence interval.

**(A) Proportion of Viremic Piglets per Litter**									
**Treatment Group**	**No. of Litters**	**Min.**	**Max.**	**Median**	**Mean**	**STD**	**95% CI**		***p***
1B-CP	16	31	100	79.4	75.8	20.3	64.9	86.6	0.005
2B-PRRSV1+PPV1	15	0	100	37.5	41.3	33.4	22.8	59.9	
3B-SC	5	0	0	0.0	0.0	0.0	0.0	0.0	---
**(B) Piglet Viral Load (log10 GE/mL)**									
**Treatment Group**	**No. of Piglets**	**Min.**	**Max.**	**Median**	**Mean**	**IQR**	**95% CI**		***p***
1B-CP	244	0	10	6.7	5.3	5.2	5.9	7.1	0.0025
2B-PRRSV1+PPV1	221	0	9.9	0.0	2.6	6.1	0.0	0.0	
3B-SC	76	0	0.0	0.0	0.0	0.0	0.0	0.0	---

**Table 3:** Proportion (%) of piglets with at least one clinical finding from farrowing to day 20 of life in Experiment B. Differences between treatment groups were tested by the Wilcoxon Mann–Whitney test. Min. = minimum value, Max. = maximum value, STD = standard deviation, CI = confidence interval.

Treatment Group	No. of Litters	Min.	Max.	Median	Mean	STD	95% CI		*p*
1B-CP	16	75	100	100	92.0	9.6	86.9	97.1	≤0.0001
2B-PRRSV1+PPV1	15	0	100	33	38.6	29.7	22.2	55.0	
3B-SC	5	0	40	20	21.3	14.5	3.4	39.3	

**Table 4:** Body weight and average daily weight gain (ADWG) (kg) of piglets. Least squares means (LSM) of the groups and differences between LSM with 95% CI were derived from the analysis of variance (ANOVA). The weight at farrowing was used as a covariate for the corresponding analysis of body weight at day 20 post-farrowing and ADWG.

	Treatment Group	No. of Animals	Mean	LSM	95% CI LSM		*p*
**Weight at Farrowing**	1B-CP	243	1.06	1.08	1.00	1.16	0.0972
	2B-PRRSV+PPV1	236	1.15	1.17	1.09	1.25	
	3B-SC	76	1.37	---	---	---	---
**Weight at 20 Days Post-Farrowing**	1B-CP	82	3.95	4.10	3.57	4.62	0.0026
	2B-PRRSV+PPV1	152	5.10	5.14	4.77	5.51	
	3B-SC	66	4.93	---	---	---	---
**ADWG**	1B-CP	82	0.14	0.14	0.12	0.17	0.0026
	2B-PRRSV+PPV1	152	0.19	0.20	0.18	0.21	
	3B-SC	66	0.18	---	---	---	---
